# Proteinase-activated receptor-2: two potential inflammatory mediators of the gastrointestinal tract in Atlantic salmon

**DOI:** 10.1186/1476-9255-5-18

**Published:** 2008-10-23

**Authors:** Jim Thorsen, Einar Lilleeng, Elin Christine Valen, Åshild Krogdahl

**Affiliations:** 1 Aquaculture Protein Centre, Basic Science and Aquatic Medicine, Norwegian School of Veterinary Science, Oslo, Norway

## Abstract

Proteinase-activated receptor 2 (PAR-2), activated by trypsin and other serine proteinases, is a key initiator of inflammatory responses in the intestine of mammals. Atlantic salmon fed diets with standard qualities of soybean meal (SBM) show enteritis of the distal intestine as well as increased activity of trypsin in both luminal contents and wall tissue. Luminal trypsin activity may possibly be involved in immune related disorders of the intestine also in Atlantic salmon via activation of PAR 2. In the present study our aim was to investigate if PAR-2 play a role in SBM induced enteritis. We performed multiple alignments based on nucleic acid sequences of PAR-2 from various animals available from public databases, and designed primers for use in cloning of the Atlantic salmon PAR-2 transcript. We further cloned and characterized the full length sequence of Atlantic salmon PAR-2 and investigated the expression in both early and chronic stages of SBM induced enteropathy. Two full length versions of PAR-2 cDNA were identified and termed PAR-2a and PAR-2b. Expression of the two PAR-2 transcripts was detected in all 18 tissues examined, but most extensively in the intestine and gills. A significant up-regulation in the distal intestine was observed for the PAR-2a transcript after 1 day feeding diets containing SBM. After 3 weeks of feeding, PAR-2a was down-regulated compared to the fish fed control diets. These findings may indicate that PAR-2a participates in inflammatory responses in both the early and later stages of the SBM enteropathy. In the chronic stages of the enteropathy, down-regulation of PAR-2a may indicate a possible desensitization of the PAR-2a receptor. Expression of PAR-2b was not altered in the first 7 days of SBM feeding, but a significant up regulation was observed after 3 weeks, suggesting a putative role in chronic stages of SBM induced enteritis. The expression differences of the two PAR-2 transcripts in the feed trials may indicate that they have different roles in the SBM induced enteritis.

## Introduction

By ingesting feed, the gastrointestinal tract (GI tract) is presented to food components and microorganisms carried with the feed exposing the organism to allergens and pathogens that can cause disease and hence affect animal welfare. The GI tract is one of a few major entry points for microorganisms and pathogens, and hence, animals have well developed physical and chemical barriers in combination with an effective mucosal immune system [1]. The mucosal and chemical barriers can be breached by microorganisms and pathogens, and once breached, circulating innate immune cells will form a second important basis for defence. The gut immune apparatus represents therefore a major element in the defence of an animal, and is considered the largest immunological organ in man [2]. A well functioning immune apparatus in the gastrointestinal tract is therefore of utmost importance for the function and wellbeing of all animals.

### SBM induced enteritis

Standard qualities of soybean meal (SBM), the most important and cheapest protein rich feedstuff on the world market, can only be used at limited levels in salmonid diets because it challenges the gut immune systems and fish health. Salmon, when fed diets with standard qualities of SBM develop an inflammation like condition (enteritis) in the distal intestine characterized by inflammatory infiltrate in the intestinal mucosa, atrophy of primary and secondary mucosal folds and decrease of epithelial vacuolization [3-5]. A previous study investigating the development of the enteritis using a 33% SBM feed showed minor changes in intestinal histology in some of the samples after two days of feeding [3]. After 7 days of feeding SBM the fish displayed all the signs present in the fully developed condition including increased width and marked reduction in height of simple mucosal folds as well as increased cell infiltration of the lamina propria [3]. Further, upon feeding SBM for 14–21 days the enteritis was fully developed in all the fish examined [3]. The SBM induced enteritis may also be a key factor in the decrease in growth as well as nutrient digestibility and absorption observed at higher inclusion levels [6-9] and has been suggested to have a negative effect on disease resistance [10]. Salmonids fed diets with standard qualities of SBM show elevated activity of trypsin-like enzymes [11,12] suggesting that trypsin might be involved in the development of SBM induced enteritis. Further, studies with partly fractionated soybean extracts have shown that the feed substances participating in the enteropathy in salmon are soluble in alcohol [13,14]. Later studies has suggested that saponins, present in the soy alcohol extract, is a compound that may cause some, but not all, of the intestinal alterations seen in Atlantic salmon fed soybean meal [15]. Saponins are known to increase permeability of intestinal tissue and thereby increase exposure to immune stimulants [16]. But still, more than 18 years after the SBM induced enteritis was reported, the causative molecular agents present in SBM responsible for the pathogenesis have not been identified.

### PAR-2 receptors and inflammation

Several studies in mammalian species have shown that activation of cell surface receptors termed proteinase-activated receptors (PARs) are key activators of inflammatory responses in a wide range of tissues including the gastrointestinal tract (GI) and airways [17-23]. These cell surface receptors are G-protein coupled and belongs to a family of seven transmembrane receptors that can be activated by serine proteinases, such as trypsin. So far, four proteinase-activated receptors have been cloned and studied in man [24-27], but to our knowledge no proteinase-activated receptors have been studied in teleost fish. Activation of PAR-2 (proteinase-activated receptor 2) in mammals is achieved by proteolytic cleavage of an extracellular peptide sequence hereby exposing an N-terminal tethered ligand domain that binds to and activates the receptor [28,29]. Upon activation of the PAR-2 receptor in mammals, the receptor is internalized and targeted to lysosomes for degradation [30,31]. To resensitize the cells from the irreversible receptor cleavage new receptors are mobilized by large Golgi stores as well as synthesis of new receptors [30]. The PAR-2 receptor has been detected in various diverse tissues such as brain, eye, airway, heart, GI tract, pancreas, kidney, liver, prostate, skin and in cells such as epithelial cells, endothelial cells, as well as in immune cells such as T-cells, neutrophils, mast cells and eosinophils [32,33]. Some of the PAR-2 mediated effects on leukocytes involve leukocyte rolling and adhesion [34] as well as leukocyte migration in vivo [35]. Activation of PAR-2 by serine proteinases have been shown, in vitro, to stimulate bone marrow progenitor cells to develop into dendritic cells [36]. Hence, serine proteinases might participate in adaptive immune responses in vivo. Activation of PAR-2 by trypsin in luminal colonocytes in mice affect the permeability and hence could play an important role in pathogenesis of different mammalian gastrointestinal disorders [22]. In humans, elevated colonic luminal serine proteinase activity of irritable bowels syndrome (IBS) patients has been suggested to involve PAR-2 activation and mediate epithelial barrier dysfunction and pathogenesis of IBS [37].

Mechanisms of immune responses in fish, whether stimulated by dietary components or pathogens are not well described. A better understanding of these responses is expected to lead the way to develop healthier and more productive diets and found the basis for improvements in disease prevention and treatments.

The reported importance of PAR-2 activation in mammalian inflammatory diseases motivated the cloning, sequencing and expression analysis of the PAR-2 transcript in Atlantic salmon fed SBM diets. The data presented indicates a possible role of PAR-2 as a mediator of inflammatory responses in the distal intestine of Atlantic salmon.

## Materials and methods

### Experimental animals

In order to study the impact of diets containing SBM on the mRNA expression of PAR-2, samples from both a short term trial (1–7 days) and a long term trial (21 days) were collected.

### Short term trial

Farmed Atlantic salmon (Salmobreed strain), weighing 214 g (average) on the termination of the experiment, were kept in fibreglass tanks (1 × 1 × 0.6 m, water depth 40–50 cm) containing running seawater (salinity 32–34 g L^-1^) under 24 h light conditions. The water temperature was between 8–10°C during the experimental period. During the experiment the fish were fed either a fishmeal (FM) diet or a diet containing 46% SBM (Table 1) for 1, 3 or 7 days. Prior to the feeding trial the fish were allocated in the fibreglass tanks and fed the fish meal diet for 27 days. Further details on formulation, chemical composition and production of the diets are given in [38]. The experiment was done at AKVAFORSK, The Institute of Aquaculture Research in Sunndalsøra (Norway).

**Table 1:** Diet formulation and composition of the diets, g kg^-1^.

	Short term trial		Long term trial	
	
Diet code	FM	SBM	FM	SBM
*Formulation*				
Fishmeal	794.6^a^	322^a^	700^c^	490^c^
Soybean meal	-	463^b^	-	300^d^
Starch	111	100	-	-
Wheat flour	-	-	144	100
Fish oil	87	109	150	105
Vitamin/mineral premix	7	6	5.0	3.5
*Chemical composition (DM)*				
DM	924	914	935.6	945.6
Lipid	142.5	158.5	248.9	174.2
Crude protein	629.8	465.6	510.9	519.1
Starch	110.3	116.0	-	-
Dietary fibre	29.4	110.4	-	-
Ash	131.9	81.6	116.3	108.6

^a^NorsECO (Egersund Sildeoljefabrikk AS, Egersund, Norway).

^b^Deno-Soy F^®^, soybean meal with hull that is hexane extracted and toasted (Denofa, Fredrikstad, Norway).

^c^Skretting Australia (Cambridge, TAS, Australia).

^d^Extruded soybean meal, Skretting Australia (Cambridge, TAS, Australia).

### Long term trial

Farmed Atlantic salmon with an initial weight of approximately 176 g were distributed into fibreglass tanks (1 × 1 × 0.6 m, water depth 40–50 cm) containing running sea water (5.6°C). The fish were fed either a FM diet or a diet containing 30% SBM for 3 weeks (Table 1). Before the start of the trial the salmon were fed a commercial diet (Skretting AS, Stavanger, Norway). Further details on formulation and chemical composition of the diets as well as fish and rearing conditions are given in [12]. The experiment was done at AKVAFORSK, The Institute of Aquaculture Research in Sunndalsøra (Norway).

### Collection of tissue samples

Fish were randomly selected, anesthetized in tricaine methansulphate (MS222), weighed, measured and killed with a sharp blow to the head followed by abdominal evisceration. The intestines were cleaned of all fatty tissue and intestinal content prior to collection of samples. The intestinal regions were defined as follows: the pyloric intestine (PI) included the intestine with the caeca; the mid intestine (MI) included the intestine between the most distal pyloric caecum and the appearance of transverse folds of the luminal surface and the increase in intestinal diameter; the distal intestine (DI) included the intestine between the distal end of the MI and anus.

For characterization of PAR-2a and PAR-2b mRNA expression in various tissues 300 mg of the following tissues were sampled from one fish fed a FM based diet: oesophagus, stomach, pancreas, PI, MI, DI, liver, head kidney, kidney, heart, spleen, thymus, brain, eye, gill, gonads, muscle and skin. All samples except for pancreas were stored in RNA*later*^® ^(Ambion Inc.) at -20°C until RNA isolation. Pancreas was collected as follows: approximately 300 mg of pancreatic tissue, i.e. diffuse pancreas embedded in the fatty tissue surrounding the pyloric caeca, was gently scraped off with a spatula and immediately snap frozen in liquid nitrogen then transferred to ten times the volume of RNA*later*^®^-ICE (Ambion, Inc.) and stored at -80°C until RNA isolation.

For quantification of PAR-2 mRNA expression approximately 300 mg of the DI from the short-term and the long-term trial was collected and stored on RNA*later*^® ^(Ambion Inc.) at -20°C until RNA isolation. The following diets and points of time were collected from the short-term trial: from the FM group (control fish) 2 fish were collected on day 1, 3 and 7 respectively (6 in total); from the SBM group 6 fish were collected at day 1, 3 and 7 respectively (6 fish per day). Nine DI samples were collected from the FM and SBM group respectively in the long-term trial.

### Total RNA extraction

Total RNA was isolated from oesophagus, stomach, pancreas, PI, MI, DI, liver, head kidney, kidney, heart, spleen, thymus, brain, eye, gill, gonads, and muscle using Trizol (Invitrogen Ltd, Paisley, UK) according to the manufacturer's protocol. A modified protocol was used for pancreas with three times the volume of reagents.

### First strand cDNA synthesis

cDNA was generated from five microgram of total RNA using PowerScript™ Reverse Transcriptase (BD Biosciences, Franklin Lakes, NJ, USA) according to the manufacturer's protocol, primed with a mixture of oligo dT (25 ng/μl) and random hexamer primers (2.5 ng/μl).

### Cloning and sequencing of PAR-2 mRNA sequences

Multiple DNA sequence alignments was performed from *Homo sapiens *[GenBank:NM_005242], *Danio rerio *[GenBank:XM_678622], *Hippoglossus hippoglossus *[GenBank:EB034068], *Oncorhynchus mykiss *[GenBank:BX861951] and *Xenopus laevis *[GenBank:BX850546] PAR-2 sequences using the publicly available web browser based bl2seq (Blast 2 Sequences, [39]). From these alignments we manually designed one degenerated (PAR-2R_Deg) and one regular PCR primer (PAR-2F) based on the identification of potential nucleic acid conservation of PAR-2 sequences. All PCR products amplified with Advantage 2 PCR enzyme mix (Clontech, Takara Bio Inc, Shiga, Japan) were used in a post-amplification procedure with addition of 2 U of Taq polymerase (Biotools, B&M Labs, Madrid, Spain) in 1× PCR buffer (Biotools) for 15 min at 72°C before use in TOPO TA cloning (TOPO TA Cloning^® ^Kit; Invitrogen, Carlsbad, CA, USA). The PAR-2 primers and cDNA generated from the distal intestine were used in PCR amplification using Advantage 2 PCR enzyme mix (Clontech) in a total reaction volume of 25 μl with the following cycling parameters: 35 cycles of 94°C for 30 s, 60°C for 30 s, and 72°C for 30 s. A positive PCR product of 149 bp was cloned using TOPO TA Cloning kit (Invitrogen) according to the manufacturers' instructions. From the cloning, five clones were selected and grown for 16 h at 37°C in Luria-Bertani media containing 50 μg/ml ampicilin. Plasmid DNA was isolated (E.Z.N.A plasmid miniprep kit I, OMEGA Bio-Tek, Inc, GA, USA) and sent for sequencing (GATC Biotech, Konstanz, Germany). From the PAR-2 sequence obtained we manually designed specific PCR primers unique for each PAR-2 versions for use in 5' and 3' RACE (rapid amplification of cDNA ends). mRNA was isolated from total RNA following the manufacturers instructions (MicroPoly(A)Purist™ Kit, Ambion, Austin, TX, USA), and approximately 1 μg of was used for reverse transcription using SMART™ RACE cDNA amplification Kit (Clontech). The PCR reactions were performed using the Advantage 2 PCR enzyme mix (Clontech) with the following touchdown PCR setup; 3 min at 94°C followed by: (30 s at 94°C, 3 min at 72°C) × five cycles, (30 s at 94°C, 30 s at 70°C, 3 min at 72°C) × five cycles, (30 s at 94°C, 30 s at 68°C, 3 min at 72°C) × 32 cycles. From each transformation, 8 clones were selected and grown for 16 h at 37°C in Luria-Bertani media containing 50 μg/ml ampicilin, plasmids were isolated (E.Z.N.A plasmid miniprep Kit I) and sequenced (GATC Biotech). The sequence chromatograms were imported to the free software ContigExpress (Vector NTI Advance 10, Invitrogen), trimmed for vector and RACE primer sequences and assembled into contigs.

### Quantitative real-time RT-PCR

Total RNA was extracted from DI as previously described. Prior to reverse transcription, total RNA from all samples were subjected to DNase treatment using a TURBO DNA-free™ kit (Ambion) in accordance with the manufacturer's recommendations.

First strand cDNA synthesis was performed with 0.8 μg total RNA from each sample using Superscript III (Invitrogen) and Oligo(dT)_20 _primers (Invitrogen) in accordance with the manufacturer's instructions.

Real-time RT-PCR primers for the two PAR-2 transcripts were designed based on the full-length sequence using the free available software FastPCR [40]. Real-time RT-PCR primers for the housekeeping genes were designed using Primer3 software [41]. PCR reactions were performed in a total volume of 10 μl using the LightCycler FastStart DNA MasterPLUS SYBR GREEN I kit (Roche Diagnostics) using 4.5 μl PCR-grade water, 0.5 μl of each PCR primer (10 μM), 2.5 μl (6.25 ng) cDNA template and 2 μl master mix. The following program was used: Denaturation (10 min at 95°C), amplification and quantification program repeated 40 times (10 sec. at 95°C, 15 sec. at the appropriate annealing temperature for the gene specific primers (Table 2) and 10 sec. at 72°C with a single fluorescence measurement), melting curve program (60°C to 99°C with a heating rate of 0.1°C/sec.) and cooling program down to 40°C.

**Table 2:** PCR primers used in cloning and real-time RT-PCR analysis

Gene name	Accession number	Primer name	PCR primer sequence	PCR annealing temperature (C°)	PCR product size (bp)
PAR-2	-	PAR-2F	ATCTACATGGCCAACCTGGC	58	149
PAR-2	-	PAR-2R_Deg	CAGTACAYGTTCCCGTAGAAGAA		
PAR-2a	-	PAR-2a_RACE_F1	GTCCGACCTGCTCTTTGTCATCTGGA	68	1316*
PAR-2a	-	PAR-2a_RACE_R1	TGGACTCCCCTGAAGATTGCCTACCAC		739*
PAR-2b	-	PAR-2b_RACE_F1	CTGGACACCTCTGAAGATCGCCTACCAC		1655*
PAR-2b	-	PAR-2b_RACE_R1	GCCCACCAGGACTTTACACAGCCT		687*
PAR-2a	-	PAR-2a_RT_F1	GCGCTACTGTGCCATCGTCAA	60	104
PAR-2a	-	PAR-2a_RT_R1	TGGTCATCAGCCAGACCCCCA		
PAR-2b	-	PAR-2b_RT_F1	ACGCTACTGGGGTGTGGCCCA		104
PAR-2b	-	PAR-2b_RT_R1	TGGTGGTGAGCCAGATGAAGG		
ELF-1 α	AF321836	SS-EF1-alpha F1	GTGCTGTGCTTATCGTTGCT	60	148
ELF-1 α	AF321836	SS-EF1-alpha R1	GGCTCTGTGGAGTCCATCTT		
β-actin	AF012125	SS beta-aktin F1	CAAAGCCAACAGGGAGAAGATGA	58	133
β-actin	AF012126	SS beta-aktin R1	ACCGGAGTCCATGACGATAC		
GAPDH	BU693999	GAPDH F1	AAGTGAAGCAGGAGGGTGGAA	60	96
GAPDH	BU693999	GAPDH R1	CAGCCTCACCCCATTTGATG		
18S rRNA	AJ427629	SS-18SrRNA F1	TACAGTGAAACTGCGAATGG	60	153
18S rRNA	AJ427629	SS-18SrRNA R1	GCATGGGTTTTGGGTCTG		

PAR-2: Proteinase-activated receptor-2, ELF-1 α: Elongation factor 1 alpha, GAPDH: Glyceraldehyde-3-phosphate dehydrogenase. * PCR product size after RACE amplification

For determination of the crossing point (CP) the "second derivative maximum method" measuring maximum increase rate of newly synthesized DNA per cycle was used on the basis of the LightCycler software 4.0 (Roche Diagnostics). To confirm amplification specificity the PCR products from each primer pair were subjected to melting curve analysis and manual inspection of PCR products after each run by agarose gel electrophoresis.

### Relative quantification analyzes

The relative expression ratio of target mRNAs was calculated using the LightCycler software 4.0 (Roche Diagnostics) with calibrator-normalized relative quantification and PCR efficiency correction based on a linear regression fit. RNA from tissues of a fish from the FM group was used as calibrator. Four reference genes; Elongation factor 1 alpha, Glyceraldehyde-3-phosphate dehydrogenase, 18S RNA and β-actin (Table 2) were analyzed for stability of expression in the samples intended for relative quantification analyses using the geNorm software [42]. Relative standard curves were generated on the basis of cDNA pooled from 2 samples from each diet and sample time, diluted in 5-fold or 10-fold dilution steps to cover the expected detection range of the target and housekeeping genes.

### Phylogenetic analyzes

To examine the evolutionary relationship of the cloned Atlantic salmon sequences we aligned them with published PAR-2 sequences from a set of animal species using MEGA 4 [43], and used Jalview [44] to visualize the aligned sequences. The following sequences were used; human [GenBank:NM_001992, GenBank:NM_005242, GenBank:NM_004101, GenBank:NM_003950], dog [GenBank:XM_546059, GenBank:XM_546057, GenBank:XM_844773, GenBank:XM_541962] mouse [GenBank:NM_010169, GenBank:NM_007974, GenBank:NM_010170, GenBank:NM_007975], rat [GenBank:NM_012950, GenBank:NM_053897, GenBank:NM_053313, GenBank:NM_053808], zebrafish [GenBank:XM_694943], frog [GenBank:NM_001085783, GenBank:NM_001086070]. In MEGA 4 we produced a cladogram using Neighbor-joining with the following settings; bootstrap = 10000 seed = 38877, complete deletion for gaps/missing data, Poisson correction for amino acid substitution and uniform rates among sites.

### Statistics

The Shapiro-Wilk W test was used to test conformity with the normal distribution. Student's t test was used to compare the relative expression of the respective genes between diets in the three week feed trial. To correct for multi comparisons of means, analysis of variance was followed by Tukey's Honestly Significant Difference (HSD) test. All results are presented as mean values with bars representing SEM. All tests were carried out two-tailed, with a significance level of 5%. The statistical analyses were performed using JMP 5.0.1 software package (SAS Institute Inc. Cary, NC, USA).

## Results and discussion

### Full-length cloning of PAR-2 transcripts

**Figure 1:**
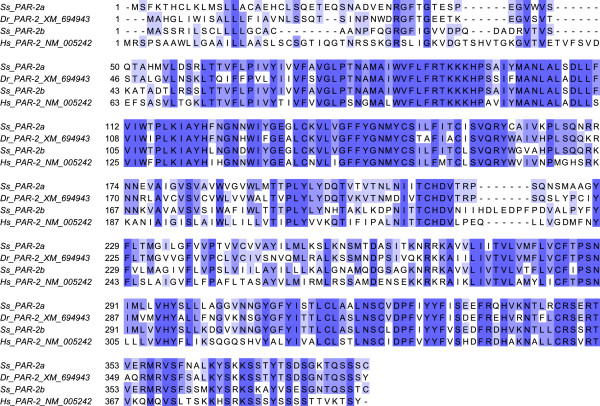
**Multiple alignments of deduced PAR-2 amino acid sequences from Atlantic salmon (Ss), zebrafish (Dr) [GenBank:**XM_694943**] and human (Hs) [GenBank:**NM_005242**] visualized using Jalview **[**44**]**.** The following percentage amino acid identity between the compared sequences is indicated with blue color, <40% identity has no color, >40% is light blue, >60% is medium dark blue, and >80% is dark blue.

**Figure 2:**
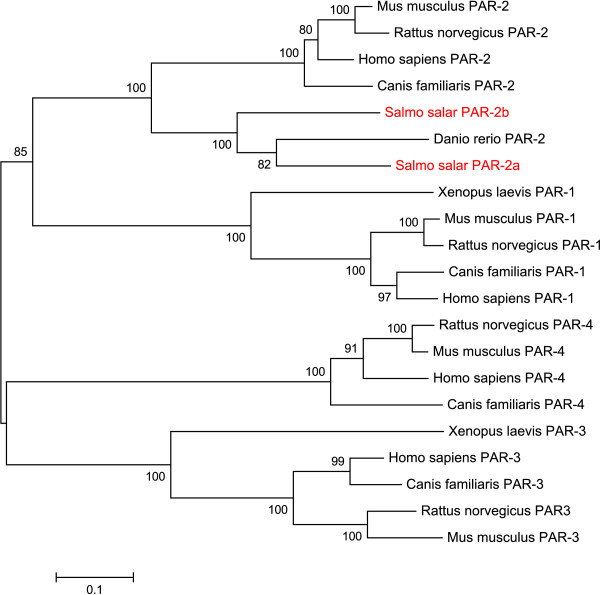
**A cladogram showing the relationship of Atlantic salmon (*Salmo salar*, in red letters), human (*Homo sapiens*), dog (*Canis familiaris*), mouse (*Mus musculus*), rat (*Rattus norvegicus*), zebrafish (*Danio rerio*) and frog (*Xenopus laevis*) proteinase-activated receptor 1–4 (PAR-1, PAR-2, PAR-3 and PAR-4).** The cladogram was created in MEGA 4 [43] using Neighbor-joining (BLOSUM62 matrix).

Using cloning and sequencing we identified two full-length PAR-2 mRNA transcripts termed PAR-2a [GenBank:FJ184031] and PAR-2b [GenBank:FJ184032] respectively, with 78% nucleic acid identity to each other in the deduced open reading frame (ORF). The large difference between the two transcripts indicate that they are likely to be derived from two genes, probably caused by divergence of the duplicated genome of Atlantic salmon [45]. Several expressed genes have previously been shown to be duplicated in Atlantic salmon [46-49], but little is known about what fraction of the reported duplicated genes are functional. Deduced amino acid similarities of the two PAR-2 receptors were compared to other species by performed multiple alignments with known PAR-2 sequences from *Homo sapiens *and *Danio rerio *(Figure 1). To visualize the phylogenetic sequence relations we produced a cladogram using Neighbour joining with BLOSUM62 matrix (Figure 2). From the alignments and the cladogram, both deduced Atlantic salmon PAR-2 sequences show similarity to other known PAR-2 sequences. However, the PAR-2a sequence show more similarity to *Danio rerio *PAR-2 sequence than to the alternative Atlantic salmon PAR-2b sequence, which may indicate PAR-2a as the putative ancestral gene. We also observed considerable differences in the first 1–45 and 200–229 aa (amino acids) of the two deduced Atlantic salmon PAR-2 proteins. These regions of the protein represent the putative N-terminal domain and extra cellular loop 2 region, both being essential in the activation of this receptor in mammals. Both deduced Atlantic salmon PAR-2 protein sequences have a serine proteinase cleavage site in the N-terminal part of the protein (Figure 1) in a comparable position of the serine proteinase site in the human PAR-2 protein.

### Expression studies of PAR-2 transcripts

**Figure 3:**
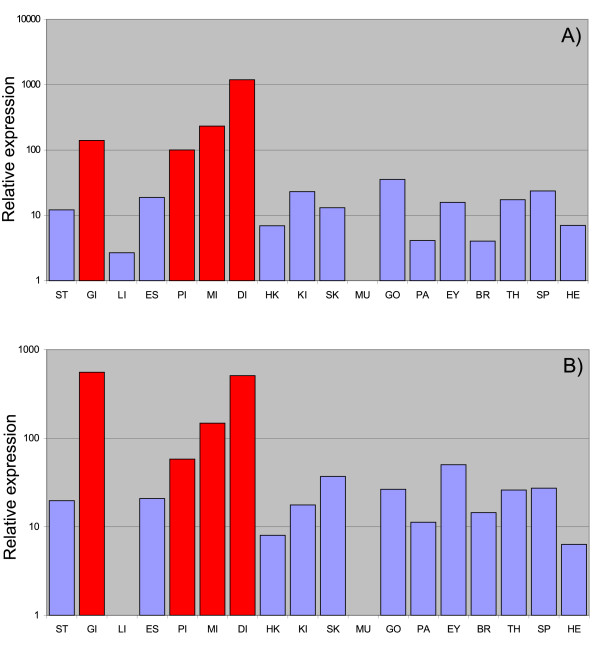
**Relative expression of PAR-2a (A) and PAR-2b (B) respectively.** Expression levels are relative to muscle tissue samples. The following tissues were examined; ST: stomach, GI: gills, LI: liver, ES: esophagus, PI: pyloric caeca, MI: mid intestine, DI: distal intestine, HK: head-kidney, KI: kidney, SK: skin, MU: muscle, GO: gonads, PA: pancreas, EY: eye, BR: brain, TH: thymus, SP: spleen, HE: heart.

Thorough testing of the PCR primer pairs with diluted plasmid templates for both PAR-2 genes in real-time RT-PCR experiments showed high specificity for the two PAR-2 transcripts (data not shown). Expression of both PAR-2 transcripts was seen in all the tissues examined, with about 10–100 fold higher expression in gills, pyloric-, mid-, and distal intestine (Figure 3). Our findings are similar to reports of high expression of PAR-2 in the colon and small intestine of man [25,50]. In man, PAR-2 has been demonstrated to mediate infiltration of leukocytes as well as hyperreactivity in allergic inflammation of the airway [23]. Hence, the high expression of the PAR-2 receptor transcripts observed in the gills of Atlantic salmon could point to analogous functions in the respiratory organ of fishes.

**Figure 4:**
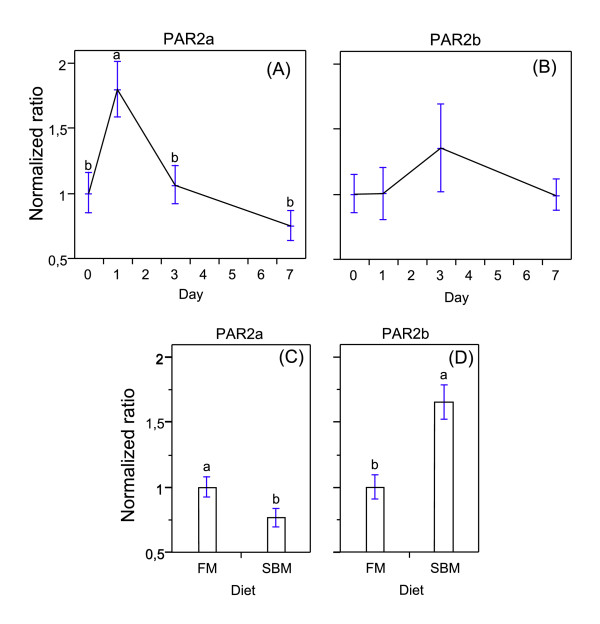
**Relative mRNA expression of PAR-2 in the distal intestine of Atlantic salmon.** The gene expression was normalized to both elongation factor 1 α and β-actin, and an average normalized ratio for each individual was calculated. The x-axis represents days after introduction to SBM and the y-axis represents the normalized ratio. Relative expression of PAR-2a (A) and PAR-2b (B) in fish fed fishmeal (FM) (n = 6) day 0 or a diet with inclusion of soybean meal (SBM) at day 1 (n = 6), at day 3 (n = 6) and at day 7 (n = 6) days. Relative expression of PAR-2a (C) and PAR-2b (D) of fish fed FM diet and diet with inclusion of SBM after 3 weeks of feeding. Error bars indicate ± S.E.M (standard error of the mean). Different lower case letters denote significant differences (P < 0.05) between the means.

Transcription level for PAR-2a showed a rapid and significant up-regulation at day 1 in fish fed diet with SBM whereas no expression differences was seen at day 3 or 7 (Figure 4). For PAR-2b there was no significant expression change during the first 7 days in fish fed diets containing SBM (Figure 4). Histopathological changes in the distal intestine of the fish fed the SBM diets showed similar features as previously described in another study [3]. No histopathological changes were observed after one day of feeding SBM, and only minor changes in some fish were seen at day three (results not shown). However, after 7 days most fish displayed the features of SBM induced enteritis (results not shown). Most fish fed SBM diet for 21 days displayed fully developed enteritis after histological investigations as been reported previously [12]. Interestingly, at three weeks feeding a diet containing SBM, a significant down-regulation was seen for PAR-2a, and a significant up-regulation was detected for PAR-2b compared to fish fed the control diet. Even though many duplicated genes in Atlantic salmon might be classified as pseudogenes, the observed response for the two PAR-2 genes may suggest involvement of both genes in the SBM induced enteritis. Overexpression of PAR-2 has been observed in biopsies from IBD (inflammatory bowels disease) patients and PAR-2 could play an important role in the development of colonic inflammation in man [20]. A pro-inflammatory role for PAR-2 was first shown in the colon of mice where acute PAR-2 activation led to an increase in epithelial permeability and bacterial translocation [17]. Patients with ulcerative colitis treated with a tryptase inhibitor shown relieved symptoms or remission of the disease [51], suggesting involvement of PAR-2 and tryptase in the gastrointestinal disease in these patients. Luminal proteinases has been demonstrated to regulate colonic paracellular permeability in mice, and that bacterial flora influences the degranulation of mucosal mast cells [22]. Further, it was suggested that the increased expression of PAR-2 observed in colonocytes is influenced by increased luminal proteinase activity rather than the release of proteinases such as tryptase [22]. Increased luminal trypsin-like activity in the distal intestine of Atlantic salmon fed diets with SBM [11,12] may suggest a similar mode of PAR-2 activation by serine proteases in fish.

In mouse colon and small intestine a higher expression of PAR-2 has been reported in the surface epithelial cells lining the upper two thirds of the villi compared to cells located in the crypt region [50]. An increased proliferative compartment length as well as lower mucosal fold height have been reported in fish fed SBM feed for three weeks or more [52]. As a consequence, the observed reduction of PAR-2a expression after 3 weeks on a SBM diet may be caused by a reduced number of proliferated cells expressing PAR-2 towards the tip of the villi compared to the control fish. If the two PAR-2 transcripts in Atlantic salmon are not expressed equally in the same cell populations, the increased expression of PAR-2b in fish fed SBM feed for 3 weeks might be caused by the number of proliferated cells or the reported leukocyte infiltrate in fish fed diets containing SBM. The expression profile of the two transcripts in different cell populations of the intestine needs to be further investigated.

Recent studies have shown that inflammation of the gut disrupts the normal microbiota and dramatically boost colonization of pathogenic bacteria in man [53,54]. An inflamed gut is therefore an open invitation to several species of pathogenic bacteria further promoting the inflammation and a factor in causing disease. The microbiota in Atlantic salmon changes upon exposure to SBM and a more diverse population of adherent bacteria has been reported after 3 weeks feeding a diet containing SBM [52]. The change of PAR-2a expression detected after one day of exposure to feed containing SBM suggest PAR-2 receptor activation and could therefore be responsible for an initiation of inflammation. Such an inflammation in concert with the new feed components could allow colonization of new bacteria and ultimately changing the normal microbiota. It is not known however if the microbiota of Atlantic salmon fed feed containing SBM is altered as early as one day after feeding, and a putative involvement of bacteria in the early phases of the development of enteritis merits further investigation.

The putative role PAR-2 seems to have in intestinal inflammation in fishes makes it a potential important marker for enteritis. Soybean meal appears as a promising tool for studies of PAR-2, intestinal inflammation responses as well as intestinal cell populations in Atlantic salmon.

## Conclusion

In our study we have demonstrated that Atlantic salmon have putative duplicated gene versions of the PAR-2 receptor. Both transcripts are highly expressed in the gastrointestinal tract and the gills. In Atlantic salmon fed inclusion levels of SBM the expression of the two PAR-2 transcripts is altered. We have shown that PAR-2a has a significant change of expression after one day of feeding diets containing SBM, but that the expression is significantly decreased in a three week feeding trial. The expression of PAR-2b did not show altered expression in the first seven days of feeding but a significant increase in expression is observed after three weeks. The altered expression of the two PAR-2 transcripts in the gut of fish fed diets containing SBM suggests that PAR-2 may have an important role in inflammation in fishes and other lower vertebrates. The identification of the PAR-2 genes in Atlantic salmon, a known initiator of inflammation in the gut of mammals, opens up for future studies to further shed light on molecular causes of the SBM induced enteritis observed in salmonids.

## Competing interests

The authors declare that they have no competing interests.

## Authors' contributions

JT planned the experiments, conducted the primer design, cloning of PAR-2 sequences, performed multiple alignment and phylogenetic analysis, participated in the real-time RT-PCR and drafted the manuscript. ECV isolated RNA, performed real-time RT-PCR and participated in the sampling. EL contributed in the real-time RT-PCR, in the sampling and in the drafting of the manuscript. ÅK participated in the planning of the experiments, drafting of the manuscript and contributed to the intellectual content.
